# Supplementary material for: Atlantic walrus signal latitudinal differences in the long-term decline of sea ice-derived carbon to benthic fauna in the Canadian Arctic
Source: Proc Biol Sci. 2020 Dec 9;287(1940):20202126. doi: 10.1098/rspb.2020.2126 (PMC7739943; doi:10.1098/rspb.2020.2126)
Supplement: Supplementary Information 1 [file rspb20202126supp1.docx]

**Title:** Atlantic walrus signal latitudinal differences in the long-term decline of sea ice-derived carbon to benthic fauna in the Canadian Arctic

**Journal:** Proceedings of the Royal Society B

**Author names:** David J. Yurkowski^*1^, Thomas A. Brown^2^, Paul J. Blanchfield^1^, Steven H. Ferguson^1^

**Author affiliations:** ^1^Fisheries and Oceans Canada, Winnipeg, Manitoba R3T 2N6, Canada

^2^Scottish Association for Marine Science, Oban, UK, PA37 1QA

**Article ID:** 10.1098/rspb.2020.2126

**Dataset:** <https://doi.org/10.5061/dryad.12jm63xwj>

**Corresponding author:** David Yurkowski ([David.Yurkowski@dfo-mpo.gc.ca](mailto:David.Yurkowski@dfo-mpo.gc.ca); [dyurkowski1@gmail.com](mailto:dyurkowski1@gmail.com))

**Trophic position equations**

We estimated trophic position for each individual using three commonly-used published equations that utilize trophic-source amino acids pairs of glutamic acid (Glu) and phenylalanine (Phe) and a combination of single- and multi-trophic discrimination factors (TDF). Both amino acids are used in the most commonly applied trophic position equation of Chikaraishi et al. (2009):

$TP=1+ \left\{ \frac{\delta^{15}N_{\mathrm{Glu}}-\delta^{15}N_{\mathrm{Phe}}- \beta}{\mathrm{TDF}_{Glu-Phe}} \right\}$ (1)

where δ^15^N_Glu_ and δ^15^N_Phe_ represent the stable isotope nitrogen values of glutamic acid and phenylalanine of the consumer, β represents the difference in δ^15^N_Glu_ and δ^15^N_Phe_ of primary producers (3.4‰; McLelland & Montoya, 2002) and TDF_Glu-Phe_ represents the constant trophic discrimination factor between diet and consumer at each trophic step (7.6‰; Chikaraishi et al., 2009). Germain et al. (2013) developed a multi-TDF approach that incorporates a seal-specific TDF estimated from controlled feeding experiments on harbour seals (*Phoca vitulina*):

$TP=2+ \left\{ \frac{\delta^{15}N_{\mathrm{Glu}}-\delta^{15}N_{\mathrm{Phe}}- \mathrm{TDF}_{Glu-Phe}}{\beta} \right\}$ (2)

where most variables and values are the same as described in equation (1) except for TDF_Glu-Phe_ which is a seal-specific TDF value of 4.3‰ (Germain et al., 2013). McMahon et al. (2015, 2019) also developed a multi-TDF approach estimated from captive gentoo penguins (*Pygoscelis papua*) during a controlled feeding trial:

$TP=2+ \left\{ \frac{\delta^{15}N_{\mathrm{Glu}}-\delta^{15}N_{\mathrm{Phe}}- \mathrm{TDF}_{Glu-Phe}-\beta}{\mathrm{TDF}_{\left( Glu-Phe \right)\mathrm{average}}} \right\}$ (3)

where most variables and values are similar to equations (1) and (2) except for TDF_Glu-Phe_ which is specific to penguins (3.5‰) and an additional variable, TDF_(Glu-Phe) average_ which represents an average TDF of 6.3‰ characteristic of planktonic marine food webs (McMahon & McCarthy, 2016; McMahon et al., 2019).

For both Jones Sound and Foxe Basin Atlantic walruses, equation (1) produced lower trophic position estimates (range = 1.9 – 2.3) by approximately 1 trophic level compared to equation (2) (range = 2.8 – 3.2) and approximately 0.5 to 1 trophic level compared to equation (3) (range = 2.5 – 3.0). Trophic position estimates ranging from 1.9 – 2.3 is unrealistically low for a species known to forage upon bivalves that are primary consumers who therefore occupy a trophic position of 2. Equations that use a universal trophic discrimination of 7.6‰ (Chikaraishi et al. 2009) have typically underestimated trophic position estimates for upper trophic level predators such as large bony fish (Lorrain et al., 2015), sharks (Hussey et al., 2015), seabirds (McMahon et al., 2019) and marine mammals (Matthews & Ferguson, 2014; Matthews et al. 2020). Atlantic walrus are generally secondary consumers, and as such, equation (2) and (3), which use a multi-trophic discrimination factor, estimated more-realistic trophic positions ranging from 2.8 – 3.2 and 2.5 – 3.0, respectively. Within each equation, the estimated trophic positions of Atlantic walrus were similar between Jones Sound and Foxe Basin with no changes occurring over time.

**References**

Chikaraishi, Y., Ogawa, N. O., Kashiyama, Y., Takano, Y., Suga, H., Tomitani, A., ... & Ohkouchi, N. (2009). Determination of aquatic food‐web structure based on compound‐specific nitrogen isotopic composition of amino acids. *Limnology and Oceanography: Methods*, **7**, 740‒750.

Germain, L. R., Koch, P. L., Harvey, J., & McCarthy, M. D. (2013). Nitrogen isotope fractionation in amino acids from harbor seals: implications for compound-specific trophic position calculations. *Marine Ecology Progress Series*, **482**, 265‒277.

Hussey, N. E., MacNeil, M. A., Siple, M. C., Popp, B. N., Dudley, S. F., & Fisk, A. T. (2015). Expanded trophic complexity among large sharks. *Food Webs*, **4**, 1‒7.

Lorrain, A., Graham, B. S., Popp, B. N., Allain, V., Olson, R. J., Hunt, B. P., ... & Kaehler, S. (2015). Nitrogen isotopic baselines and implications for estimating foraging habitat and trophic position of yellowfin tuna in the Indian and Pacific Oceans. *Deep Sea Research Part II: Topical Studies in Oceanography*, **113**, 188‒198.

Matthews, C. J., & Ferguson, S. H. (2014). Spatial segregation and similar trophic-level diet among eastern Canadian Arctic/north-west Atlantic killer whales inferred from bulk and compound specific isotopic analysis. *Journal of the Marine Biological Association of the United Kingdom*, **94**, 1343‒1355.

Matthews, C. J., Ruiz‐Cooley, R. I., Pomerleau, C., & Ferguson, S. H. (2020). Amino acid δ^15^N underestimation of cetacean trophic positions highlights limited understanding of isotopic fractionation in higher marine consumers. *Ecology and Evolution*, **10**, 3450‒3462.

McClelland, J. W., & Montoya, J. P. (2002). Trophic relationships and the nitrogen isotopic composition of amino acids in plankton. *Ecology*, **83**, 2173‒2180.

McMahon, K. W., & McCarthy, M. D. (2016). Embracing variability in amino acid δ15N fractionation: mechanisms, implications, and applications for trophic ecology. *Ecosphere*, **7**, e01511.

McMahon, K. W., Michelson, C. I., Hart, T., McCarthy, M. D., Patterson, W. P., & Polito, M. J. (2019). Divergent trophic responses of sympatric penguin species to historic anthropogenic exploitation and recent climate change. *Proceedings of the National Academy of Sciences*, **116**, 25721‒25727.

McMahon, K. W., Polito, M. J., Abel, S., McCarthy, M. D., & Thorrold, S. R. (2015). Carbon and nitrogen isotope fractionation of amino acids in an avian marine predator, the gentoo penguin (Pygoscelis papua). *Ecology and Evolution*, **5**, 1278‒1290.
